# Centrosome reduction in newly-generated tetraploid cancer cells obtained by separase depletion

**DOI:** 10.1038/s41598-020-65975-1

**Published:** 2020-06-04

**Authors:** Claudia Galofré, Elena Asensio, Maria Ubach, Irianna M. Torres, Isabel Quintanilla, Antoni Castells, Jordi Camps

**Affiliations:** 1 Gastrointestinal and Pancreatic Oncology Team, Institut D’Investigacions Biomèdiques August Pi i Sunyer (IDIBAPS), Hospital Clínic de Barcelona, Centro de Investigación Biomédica en Red de Enfermedades Hepáticas y Digestivas (CIBEREHD), Barcelona, 08036 Spain; 2 Unitat de Biologia Cel·lular i Genètica Mèdica, Departament de Biologia Cel·lular, Fisiologia i Immunologia, Facultat de Medicina, Universitat Autònoma de Barcelona, Bellaterra, 08193 Spain; 3 Cell Biology of Genomes Group, National Cancer Institute, National Institutes of Health, Bethesda, MD 20817 USA

**Keywords:** Chromosome segregation, Mitosis, Centrosome, Cancer genetics

## Abstract

Tetraploidy, a common feature in cancer, results in the presence of extra centrosomes, which has been associated with chromosome instability (CIN) and aneuploidy. Deregulation in the number of centrosomes triggers tumorigenesis. However, how supernumerary centrosomes evolve during the emergence of tetraploid cells remains yet to be elucidated. Here, generating tetraploid isogenic clones in colorectal cancer and in non-transformed cells, we show that near-tetraploid clones exhibit a significant increase in the number of centrosomes. Moreover, we find that centrosome area in near-tetraploids is twice as large as in near-diploids. To evaluate whether centrosome clustering was occurring, we next analysed the number of centrioles revealing centriole amplification. Notwithstanding, more than half of the near-tetraploids maintained in culture do not present centrosome aberrations. To test whether cells progressively lost centrioles after becoming near-tetraploid, we transiently transfected diploid cells with siRNA against *ESPL1*/Separase, a protease responsible for triggering anaphase, to generate newly near-tetraploid cells. Finally, using this model, we assessed the number of centrioles at different time-points after tetraploidization finding that near-tetraploids rapidly lose centrosomes over time. Taken together, these data demonstrate that although most cells reduce supernumerary centrosomes after tetraploidization, a small fraction retains extra centrioles, potentially resulting in CIN.

## Introduction

Centrosomes are the major microtubule organizing centres in animal cells and consist of two orthogonally orientated centrioles surrounded by a protein-rich pericentriolar material (PCM). As the main microtubule organizing centre of the animal cell, centrosomes participate in the nucleation of the interphase cytoskeleton, and are responsible for establishing the metaphase plate during mitosis. Centrioles are microtubule-based structures involved in controlling polarity, proliferation, migration, signalling and cell division^1^. In cycling cells, centrioles duplicate every cell cycle during S-phase by forming a new procentriole. The centriole biogenesis is controlled at three distinct levels: a) spatial, procentriole assembly is restricted to the proximal end of the existing centriole; b) numerical, the building of each procentriole is limited to exactly one per each parent centriole; and c) temporal, centrioles are licenced for a new round of duplication once per cell cycle^2^. Centriole duplication is controlled through a centrosome-intrinsic blockade, in which duplication of the parent centriole is prevented as long as the parent and the newly-synthetized procentriole remain in a tight orthogonal association^3^. The dissolution of this linkage, known as centriole disengagement, requires the activity of the kinase PLK1 and the protease separase, which permits the reduplication of the parent centriole in the next cell cycle^4^. Recent advances in measuring the number and size of centrioles have demonstrated that centriole over-elongation in cancer cells leads to ectopic procentriole formation^5^.

The disturbance of the centrosome homeostasis represents a major misfortune in the cellular physiology. In fact, centrosome aberrations are commonly observed in tumours^6^, correlating with increased tumour aggressiveness and poor prognosis^7–9^. Centrosome alterations in human cancers are either numerical, which mostly reflect increases in the number of centrosomes (also referred as centrosome amplification or supernumerary centrosomes), or structural, comprising alterations in centriole length or in the amount of PCM. Recent work has shown that induced centrosome amplification by increasing the levels of PLK4 can promote and/or accelerate tumorigenesis in mice through the generation of aneuploidy^10,11^. In addition, the presence of extra centrosomes can increase the microtubule nucleation capacity, which promotes cell migration and invasion^12^. Similarly, structural alterations in centrosomes have also been shown to promote basal cell extrusion, permitting the dissemination of genetically unstable cells possibly providing a route for metastasis^13^.

Centrosome amplification can arise via cytokinesis failure resulting in tetraploidization, which by itself is a major cause of chromosome instability (CIN) in human tumours^14–16^. Multipolar spindles, characteristic of cells containing extra centrosomes, lead to inviable divisions due to severe levels of aneuploidy. Thus, cancer cells reorient their multiple spindles into a pseudo-bipolar array by clustering of extra centrosomes in order to efficiently segregate chromosomes in two viable daughter cells^17–19^. However, during the clustering process, lagging chromosomes are generated as a consequence of merotelic attachments during anaphase and, consequently, chromosome missegregation and CIN emerge^20–22^. Supernumerary centrosomes provide an advantage to cancer cells by acting as a source of chromosomal and genetic instability allowing karyotype evolution^23^. Nonetheless, tetraploid cells lose extra centrosomes after passages in culture^22^, and even in the case of repeated rounds of cytokinesis failure do not permit to maintain centrosome amplification over time^24^. However, whether extra centrosome loss is also occurring in tetraploid cancer cells and its kinetics remain unclear. Thus, further work is needed to fully understand centriole dynamics during the generation and establishment of tetraploid cells.

Here, we systematically analyse the number and structure of centrosomes in four isogenic models of near-diploid (2N) and near-tetraploid (4N) cells, including three colorectal cancer cell lines and the non-transformed RPE1 cell line. Our results show an enlarged centrosome area in 4N cells even though only a small amount of them display centrosome amplification. Furthermore, by interrogating the differentially expressed genes that contribute to CIN, we have identified overexpression of *ESPL1*/Separase in near-tetraploids. Finally, the generation of 4N cells, either by silencing *ESPL1*/Separase or dihydrocytochalasin B (DCB) treatment, reveals that most newly-generated 4N cells rapidly reduce extra centrosomes over time, although a small fraction retains supernumerary centrosomes, thus resulting in CIN.

## Results

### Enlarged centrosomes, indicative of centriole amplification, are observed in 4N cells

**Figure 1:**
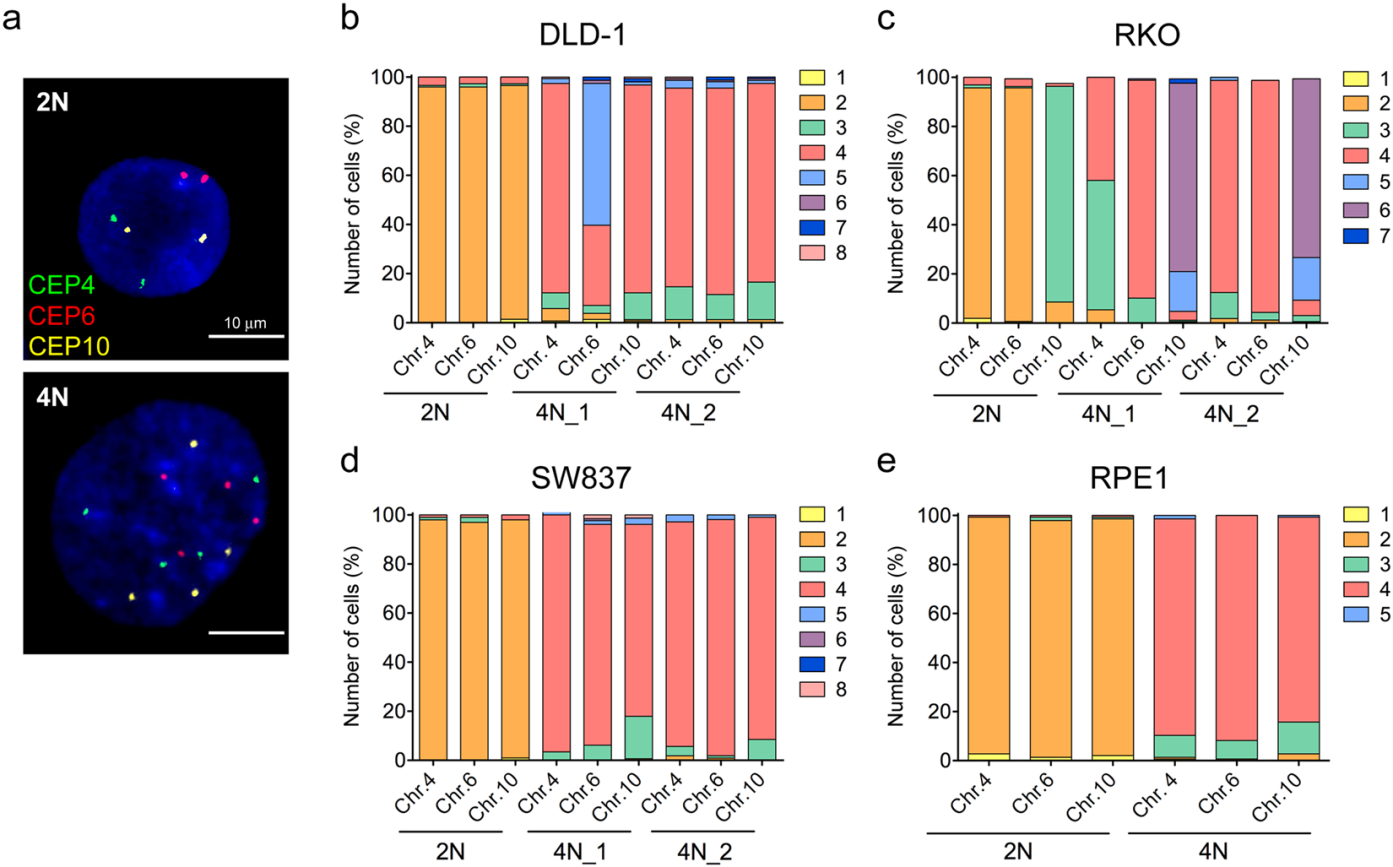
Assessment of CIN levels by FISH in 2N and 4N isogenic models. (**a**) Representative images of 2N (top) and 4N (bottom) DLD-1 isogenic clones after FISH using centromeric probes specific for chromosomes 4 (green), 6 (red) and 10 (yellow). DAPI was used for nuclear counterstaining. (**b–e**) Graphs illustrate percentage of cells with corresponding number of FISH signals for chromosomes 4, 6 and 10 for one 2N and two 4N clones of DLD-1 (**b**), RKO (**c**) and SW837 (**d**), and one 2N and one 4N RPE1 clones (**e**). A total of ~200 nuclei were analysed for each clone.

To establish a model to investigate the relationship between tetraploidy and the number and structure of centrosomes, we used 2N and 4N isogenic cells derived from the colorectal cancer cell lines DLD-1, RKO and SW837, and the non-transformed RPE1. In agreement with previous data generated in our laboratory^16^, we confirmed increased levels of CIN by fluorescence *in situ* hybridization (FISH) analysis in 4N compared to 2N cells (Fig. 1a). While 2N clones exhibited disomic content for chromosomes 4, 6, and 10 in most of the cells from all four cell lines with the exception of RKO, which presented a gain of chromosome 10 in the parental line (Figs. 1b-e), 4N clones did not only show that the majority of the cellular population doubled the amount of FISH signals for the above-mentioned chromosomes, but also a greater amount of chromosomal number variability, with a preference for chromosome losses (Fig. 1b-e). This higher degree of karyotype heterogeneity was further validated by counting metaphase spreads. In fact, modal numbers of 45 chromosomes in DLD-1, 49 in RKO, 46 in SW837 and 47 in RPE were systematically observed in 2N cells; however, 4N clones displayed a wider variability in the number of chromosomes per cell across all cell lines and modal numbers corresponded to 90 in DLD-1, 94 in RKO, 92 in SW837 and 92 in RPE1 (Supplementary Fig. 1).

As previous γ-tubulin staining indicated that 4N clones displayed a larger sub-population of cells with extra centrosomes compared to 2N clones in DLD-1 and RKO^16^, we wanted to further validate these results using pericentrin staining and including all four cell lines. The number of centrosomes in G1 phase cells was assessed by coimmunostaining of cyclin D1 and pericentrin, confirming that a significant population of cells in 4N clones displayed extra centrosomes compared to 2N clones (mean 11.39% *vs* 5.6%, ANOVA test, *P* = 0.02 for DLD-1; mean 11.63% *vs* 3.79%, ANOVA test, *P* = 0.02 for RKO; mean 12.47% *vs* 8.35*%*, ANOVA test, *P* = 0.02 for SW837; and mean 11.95% *vs* 6.17%, ANOVA test, *P* = 0.03 for RPE1) (Supplementary Fig. 2). Nevertheless, when we looked at multipolarity in near-tetraploid anaphase cells, we found that the number of 4N cells exhibiting multipolar mitoses was not significantly different from 2N cells (mean 6.21% *vs* 5.72%, *P* = 0.89 for DLD-1 cells; mean 7.12% *vs* 1.96%, *P* = 0.35 for RKO cells; mean 1.30% *vs* 1.28%, *P* = 0.99 for SW837 cells; mean 1.32% *vs* 1.08%, *P* = 0.85 for RPE1 cells) (Supplementary Fig. 2).

**Figure 2:**
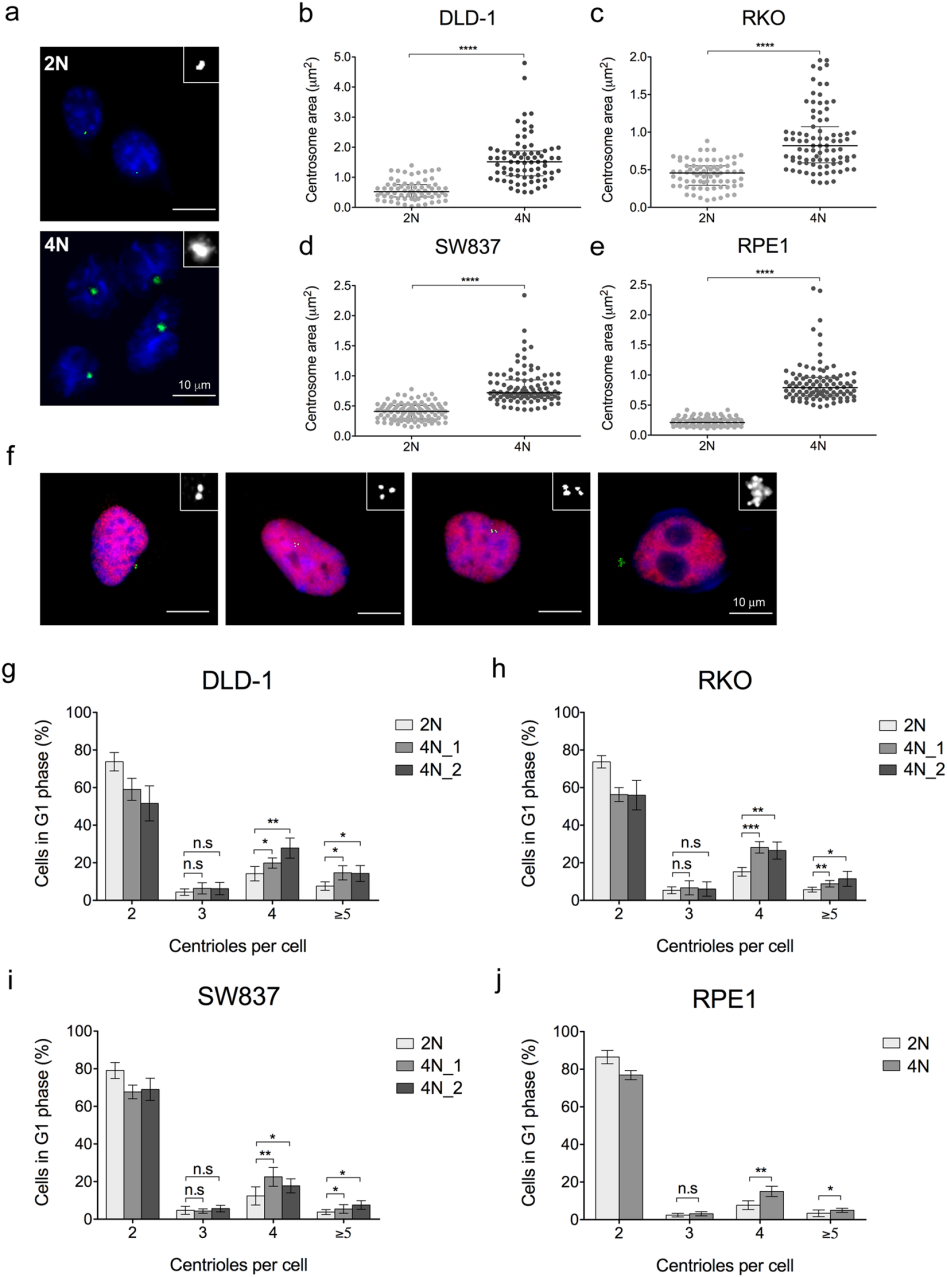
Enlarged centrosomes, indicative of centriole amplification events, are observed in 4N cells. (**a**) Representative images of 2N (top) and 4N (bottom) cells after pericentrin (green) immunostaining. DAPI was used for nuclear counterstaining. Inserts represent image amplifications in black and white of the green channel (anti-pericentrin). (**b–e**) Dot plots depicting centrosome area (µm^2^) in individual cells from one 2N and one 4N clone for DLD-1 (**b**), RKO (**c**), SW837 (**d**) and RPE1 (**e**) cell lines. Black lines denote median with interquartile range centrosome area for each clone (n = 100 centrosomes/clone). (**f**) Representative images of 4N cells immunostained for centrin3 (green) and cyclin D1 (red) showing increasing numbers of centrin3 dots from left to right: 2 centrioles (left side), 3 centrioles (middle-left), 4 centrioles (middle-right) and >5 centrioles (right side). DAPI was used for nuclear counterstaining. Inserts represent image amplifications in black and white of the green channel (anti-centrin3). (**g–j**) Bar plots showing percentage of cells in G1 phase with corresponding number of centrioles per cell for one 2N and two 4N clones of DLD-1 (**g**), RKO (**h**) and SW837 (**i**) cell lines, and for one 2N and one 4N RPE1 (**j**) clones. A total of ~750 cells were analysed for each clone. Data are reported as means ± SD. * represents *P* < 0.05, **P < 0.01, ***P < 0.001, and ****P < 0.0001 (n.s., not significant).

Besides enumerating centrosomes, we also sought to determine their size. As 4N cells should present two centrosomes in G1 phase, the observed high frequencies of cells with one centrosome could indicate that 4N cells were extruding one centrosome from the cell or that centrosomes remained tightly joined together as a result of the clustering process, thus only one signal by pericentrin staining was detected. Abnormally large centrosomes, i.e. showing a pericentrin signal larger than twice their usual size in its diploid counterpart, were observed in 4N cells (mean 1.47 μm^2^ *vs* 0.58 μm^2^, *P* < 0.0001 for DLD-1 cells; mean 0.97 μm^2^ *vs* 0.44 μm^2^, *P* < 0.0001 for RKO cells; mean 0.81 μm^2^ *vs* 0.41 μm^2^, *P* < 0.0001 for SW837 cells; mean 0.86 μm^2^ *vs* 0.22 μm^2^, *P* < 0.0001 for RPE1 cells) (Fig. 2a-e).

To evaluate whether these abnormally large pericentrin signals corresponded to centriole amplification events rather than a PCM matrix enlargement, immunostaining using antibodies against cyclin D1 and centrin3, which labels single centrioles, was performed (Fig. 2f). We found that the number of cells in G1 phase with ≥4 centrioles per cell was significantly higher in 4N cells compared to 2N cells (mean 38.33% *vs* 21.80%, *P* < 0.0001 for DLD-1 cells; mean 37.5% *vs* 20.89%, *P* < 0.0001 for RKO cells; mean 26.64% *vs* 15.87%, *P* < 0.0001 for SW837 cells; mean 19.97% *vs* 11.11%, *P* < 0.01 for RPE1 cells) (Fig. 2g-j). Intriguingly, despite our analysis revealed that the number of centrioles per cell was significantly higher in 4N cells, more than half of the 4N population (55.34% of DLD-1 cells, 56.14% of RKO cells, 68.49% of SW837 cells, 76.89% of RPE1 cells) presented two centrioles at G1 phase in average across all cell lines, suggesting that the frequency of 4N cells with four or more centrioles was much lower than the expected considering the potential occurrence of centrosome clustering.

### Transcriptional profiling of 4N cells reveals deregulation of genes associated with CIN

**Figure 3:**
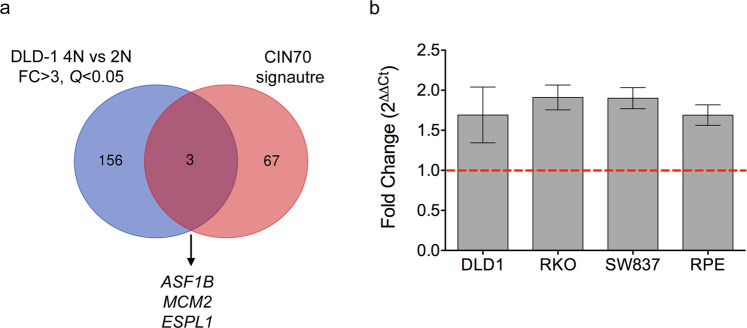
Deregulation of genes associated with CIN in 4N cells. (**a**) Venn diagram of differentially expressed genes contributing to genome doubling. Blue represents the 159 most differentially expressed genes between 4N and 2N clones obtained from genome-wide transcriptome profiling (fold-change >3 and *Q* < 0.05), and red represents the 70 top-ranking genes with the highest CIN score^25^. (**b**) Graph showing the fold-change assessed by real time RT-qPCR of the gene *ESPL1* in DLD-1, RKO, SW837 and RPE1 4N cells compared to their 2N counterparts. *GAPDH* was used as a housekeeping gene. Dashed red line represents the cut-off for overexpression.

In order to assess differentially expressed genes contributing to genome doubling and potentially involved in CIN and centrosome aberrations, we utilized genome-wide transcriptome profiling data of five 2N and ten 4N DLD-1 clones previously obtained in our laboratory and publicly accessible at the Gene Expression Omnibus (accession number GSE81395). Then, we overlapped the most differentially expressed genes between 4N and 2N clones [fold change (FC) > 3, false discovery rate (FDR) *Q* < 0.05], which generated a list of 159 genes, with the CIN70 signature (list of 70 top-ranking genes with the highest CIN score)^25^ (Supplementary Table 1). The result of this intersection provided three genes commonly shared in the two gene lists: *ASF1B* (FC = 4.28, *Q* = 0.0026), *MCM2* (FC = 3.75, *Q* = 0.0028) and *ESPL1* (FC = 3.15, *Q* = 0.0285) (Fig. 3a). The latter one encodes the separase, a protease required for sister chromatid separation during mitosis in human cells and involved in the proximity control of the two centrioles in the centrosome^26^. Overexpression of *ESPL1*/Separase was confirmed at the mRNA level in the 4N clones across the four cell lines (Fig. 3b).

### Silencing of *ESPL1* induces tetraploidization

**Figure 4:**
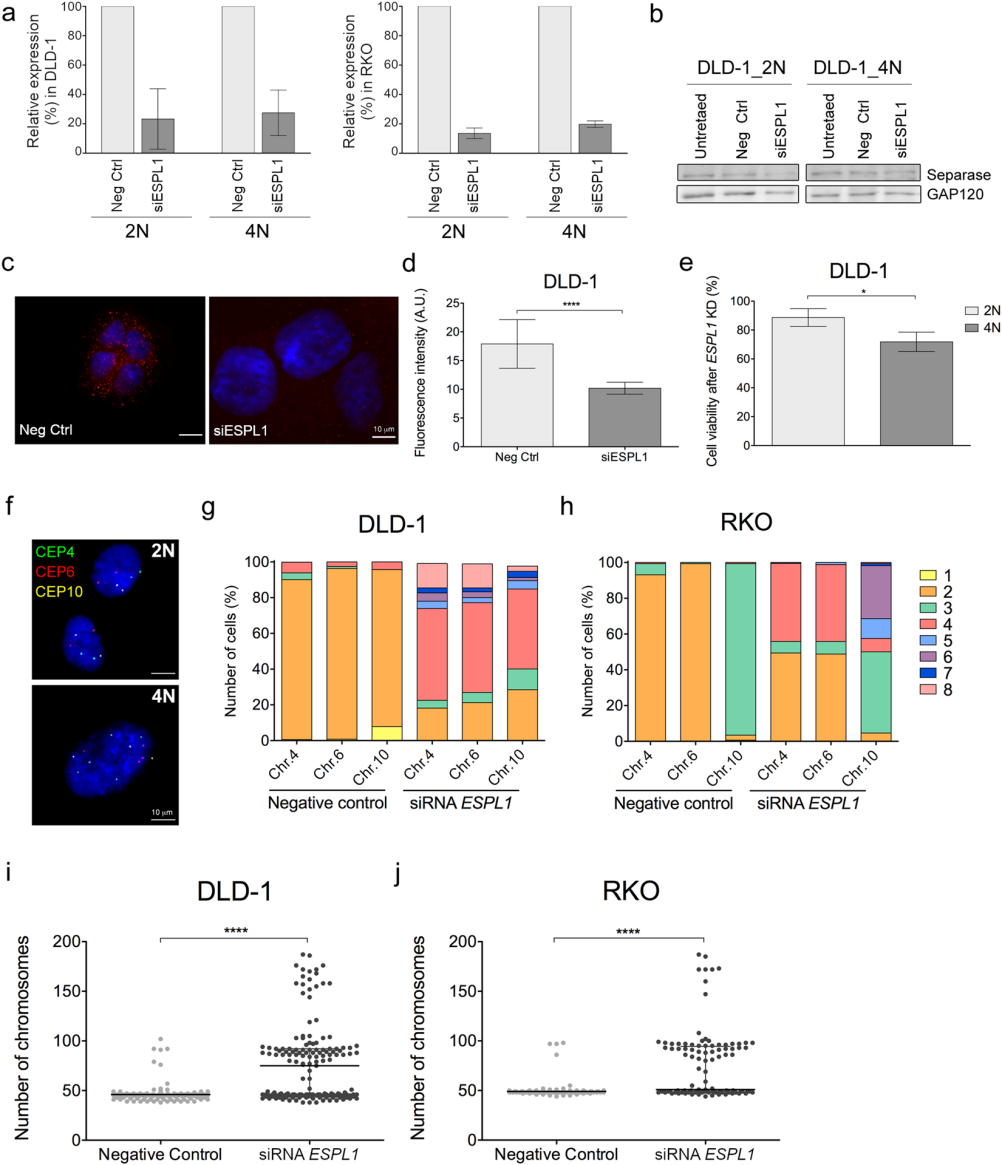
Silencing of *ESPL1* induces tetraploidization. (**a**) Relative expression (%) of *ESPL1* after transient transfection with negative control and *ESPL1* siRNAs in 2N and 4N DLD-1 (left) and RKO (right) cells. *GAPDH* was used as a housekeeping gene for normalization. Data are reported as means ± SD (n = 4 independent experiments/cell line). (**b**) Immunoblot showing decreased expression of separase after inducing gene silencing by siRNA against *ESPL1* for 96 h. GAP120 was used as protein loading control. Blotting for separase and the loading control GAP120 was performed from the same gel after stripping the membrane. (**c**) Representative images of immunofluorescence against separase (red) comparing negative control (left) and cells treated with siRNA against *ESPL1* (right). DAPI was used for nuclear counterstaining. (**d**) Bar plot showing the quantification of immunofluorescence staining in interphase nuclei. A minimum of 40 fields of view from two different slides of each condition (corresponding to a minimum of 150 nuclei) were analysed using ImageJ. Data are reported as mean ± SD. (**e**) Graph depicting significantly greater cell viability reduction in 4N compared to 2N DLD-1 cells after transient transfection with *ESPL1* siRNA. Non-specific siRNA-treated cells were used as a negative control. Data are expressed as means ± SD (n = 4 independent experiments) (**f**) Representative FISH images of 2N DLD-1 cells before (top) and after (bottom) transient transfection with siRNA against *ESPL1*. FISH assays include centromeric probes for chromosome 4 (green), 6 (red) and 10 (yellow). DAPI was used for nuclear counterstaining. (**g,h**) Stacked bar graphs illustrate the percentage of cells with the corresponding number of signals for chromosomes 4, 6 and 10 for 2N DLD-1 (**g**) and RKO (**h**) cells transfected either with negative control or *ESPL1* siRNA. More than 500 nuclei were analysed for each condition and cell line. i,j) Dot plot depicting number of chromosomes in individual cells from DLD-1 (**i**) and RKO (**j**) cells transfected either with negative control or *ESPL1* siRNA. Black lines denote median chromosome number with interquartile range for each condition (n > 100 metaphases/condition). * represents *P* < 0.05 and ****P < 0.0001.

Since 4N cells showed overexpression of *ESPL1*, we transiently reduced the expression of *ESPL1* to investigate whether 4N cells displayed less tolerance to the decrease of separase compared to 2N cells. First, gene silencing was confirmed in DLD-1 and RKO clones at the mRNA level (Fig. 4a). In addition, in DLD-1 clones gene silencing was also validated at the protein level by western blot and immunofluorescence (Fig. 4b-d and Supplementary Fig. 3). Next, we conducted cell viability assays, which showed a reduced cell viability in separase-depleted DLD-1 cells compared to negative control transfected cells (Fig. 4e). Moreover, this assay also revealed a significant decrease of cell viability in separase-depleted DLD-1 4N clones compared to their 2N counterparts (*P* < 0.05).

Because separase has been shown to be required for sister chromatid separation in human cells^27^, we assessed the ploidy status of DLD-1 and RKO 2N cells at 72 h post transfection with siRNA against *ESPL1* by FISH (Fig. 4f). Our analysis showed that *ESPL1* silencing resulted in an abundant formation of polyploid cells in both cell lines (76.81% in DLD-1 and 50.58% in RKO), with tetraploid content being the most frequently represented (Fig. 4g,h). These results were further validated by counting chromosomes on metaphase spreads demonstrating that 58.5% of the cellular population in DLD-1 and 46.8% in RKO became near-tetraploid after *ESPL1* silencing (Fig. 4i,j). *ESPL1*-depleted DLD-1 2N cells are referred to as newly-generated 4N cells.

### Time-course analysis reveals centriole reduction in newly-generated 4N cells

**Figure 5:**
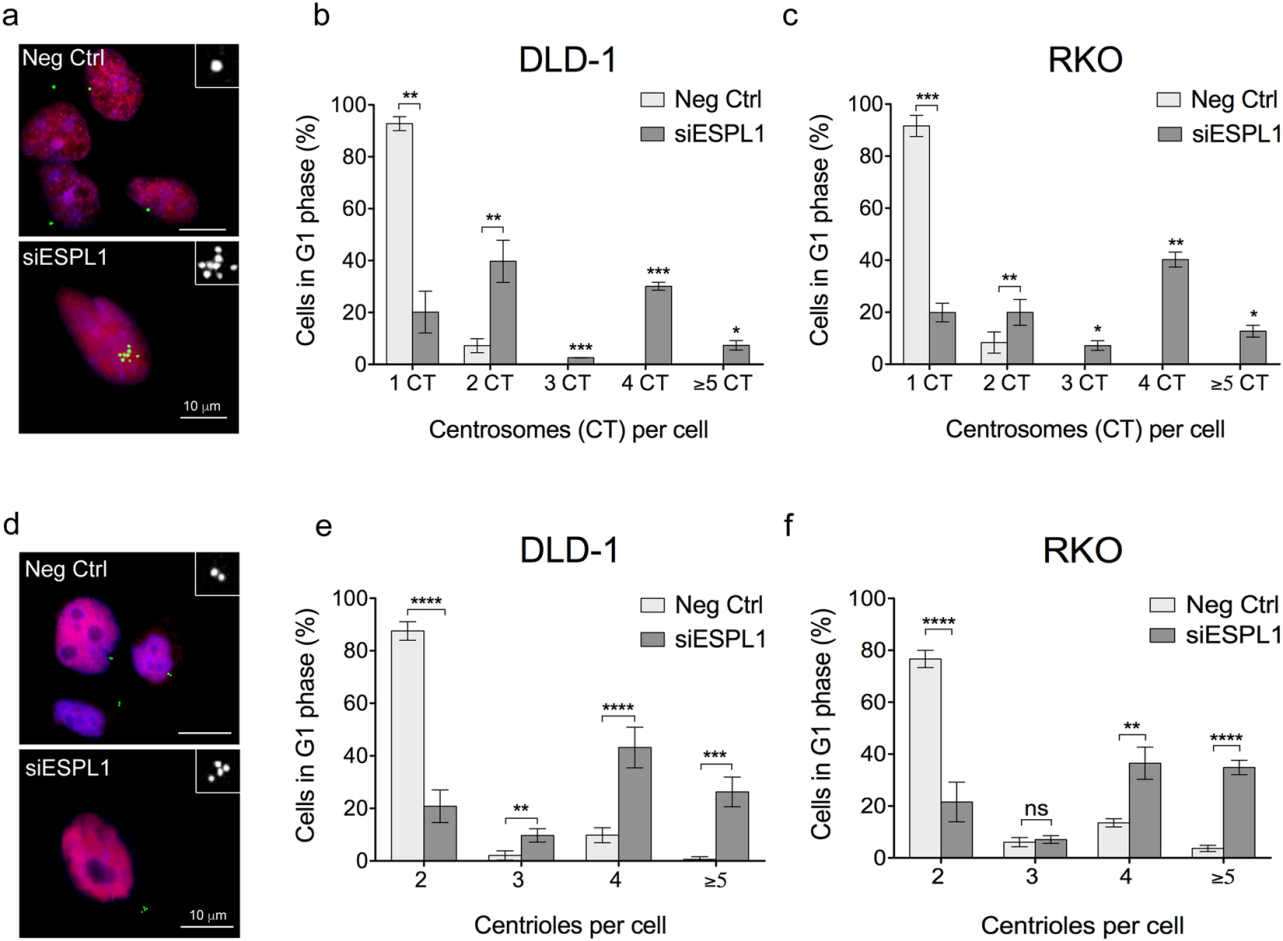
Centrosome amplification in cells after transiently silencing *ESPL1*. (**a**) Representative images of 2N DLD-1 cells immunostained with pericentrin (green) and cyclin D1 (red) antibodies. Cells transfected with negative control siRNA (top) and with siRNA against *ESPL1* (bottom). DAPI was used for nuclear counterstaining. Inserts represent image amplifications in black and white of the green channel (anti-pericentrin). (**b,c**) Graphs depicting centrosome number in individual DLD-1 (**b**) and RKO (**c**) cells transfected either with negative control or *ESPL1* siRNA. A total of ~250 cells were analysed for each condition for both cell lines. (**d**) Centrin3 (green) and cyclin D1 (red) co-immunostaining representative images of DLD-1 2N cells transfected with negative control or *ESPL1* siRNA. While negative control transfected cells display two centrioles (top), a cell transfected with *ESPL1* siRNA shows four centrioles (bottom). DAPI was used for nuclear counterstaining. Inserts represent image amplifications in black and white of the green channel (anti-centrin3). (**e,f**) Graphs showing the number of centrioles in 2N DLD-1 (**e**) and RKO (**f**) cells transfected with negative control or *ESPL1* siRNA. A total of ~750 cells were analysed for each condition of DLD-1, and ~500 cells for each condition of RKO. Data are reported as means ± SD. * represents *P* < 0.05, **P < 0.01, ***P < 0.001, and ****P < 0.0001 (n.s., not significant).

To test whether newly-generated 4N cells after *ESPL1* silencing reproduced the same levels of centrosomes amplification that was observed in established 4N clones, first we coimmunostained with pericentrin and cyclin D1 at 72 h post-transfection with siRNA against *ESPL1* (Fig. 5a). Results showed supernumerary centrosomes, i.e. two or more, in *ESPL1*-depleted DLD-1 2N cells in comparison to those transfected with negative control (mean 79.84% *vs* 7.25%, *P* < 0.01 for DLD-1 cells; mean 80.12% *vs* 8.38%, *P* < 0.001 for RKO cells) (Fig. 5b-c). The abundant number of cells with extra centrosomes in the newly-generated 4N cells is in contrast to the previously identified phenotype in established near-tetraploid cells. Moreover, it is worth noting the presence of cells with more than two centrosomes which was not found in established near-tetraploid clones (Supplementary Fig. 2). To assess whether the number of extra centrosomes corresponded with an increase in centriole number, cyclin D1 and centrin3 co-immunostaining was performed in DLD-1 and RKO cells at 72 h post- transfection (Fig. 5d). Results revealed an overall increased number of cells with ≥4 centrioles in *ESPL1*-depleted 2N cells compared to negative control transfected cells (mean 69.46% *vs* 10.36%, *P* < 0.0001 for DLD-1 cells; mean 71.37% *vs* 17.23%, *P* < 0.01 for RKO cells) (Fig. 5e-f). Additionally, these data also indicated that newly-generated 4N cells as a result of *ESPL1* silencing displayed a larger subpopulation of cells with extra centrioles compared to established 4N clones (mean 69.46% *vs* 38.33%, *P* < 0.0001 for DLD-1 cell line; mean 71.37% *vs* 37.50%, *P* < 0.0001 for RKO cell line) (Fig. 2g-j).

**Figure 6:**
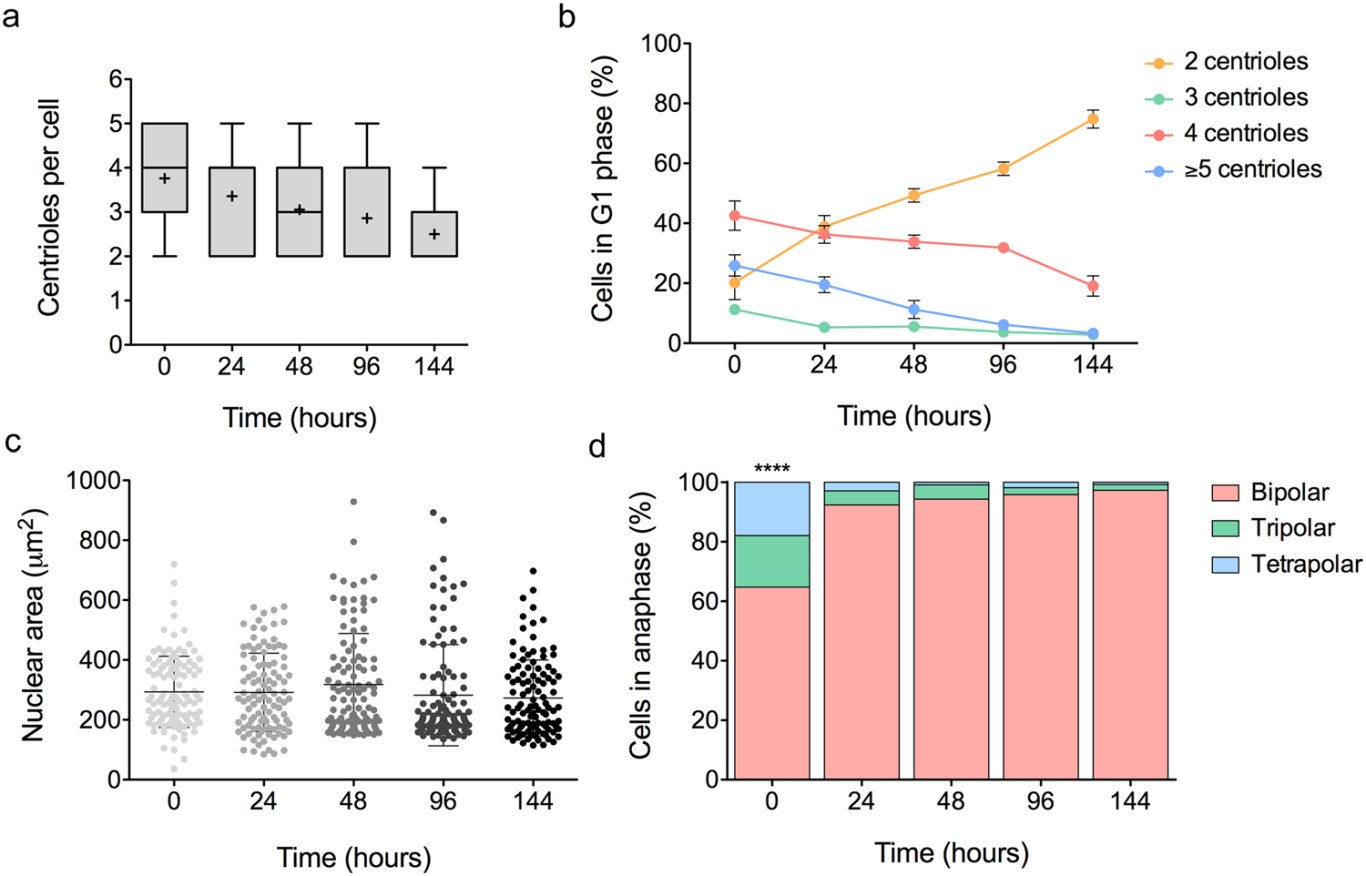
Centriole reduction in newly-generated 4N cells. (**a**) Box and whisker plot depicting the number of centrioles per cell at 0, 24, 48, 96 and 144 h after tetraploidization. More than 500 nuclei were analysed for each time-point. Data are reported in boxes which extend from the 25th to 75th percentiles and whiskers from the smallest value up to the largest. Black lines denote median (4 at 0 h, 4 at 24 h, 3 at 48 h, 2 at 96 h, and 2 at 144 h) and “+” indicates the mean. Differences between time-points are significant (*P* < 0.0001). (**b**) Plots showing time-course experiments to characterise the number of centrioles. A reduction of centriole number throughout the different time-points after tetraploidization was observed. A total of ~500 nuclei were analysed for each time-point. Data are reported as means ± SD. (**c**) Dot plot depicting the nuclear area (µm^2^) at different time-points after tetraploidization. No significant differences were found between different time-points. Black lines denote mean ± SD (n = 100 nuclei/time-point). (**d**) Stacked bar graph showing the frequency of bipolar and multipolar anaphases. Note that significant differences were only found at 0 h after siRNA washout. Data are reported as mean (n = 200–400 anaphases/time-point). **** represent *P* < 0.0001.

To study centriole dynamics and determine whether the newly-generated 4N cells were spontaneously losing their extra centrioles over time, we assessed the number of centrioles in G1 phase at different time-points (0, 24, 48, 96, and 144 h) post-*ESPL1* siRNA transfection. We found that the mean number of centrioles in DLD-1 cells gradually decreased after tetraploidization (mean 3.75 at 0 h, mean 3.37 at 24 h, mean 3.07 at 48 h, mean 2.86 at 96 h, mean 2.51 at 144 h after tetraploidization; ANOVA test, *P* < 0.0001) (Fig. 6a). Throughout the different time-points after siRNA washout, the mean frequency of cells with ≥4 centrioles displayed also a significantly decrease (mean 69.46% at 0 h, mean 55.81% at 24 h, mean 45.11% at 48 h, mean 38.06% at 96 h, mean 22.35% at 144 h; ANOVA test, *P* < 0.0001) (Fig. 6b). To test whether the loss of extra centrosomes in DCB-induced newly-generated 4N cells displayed similar kinetics, cyclin D1 and centrin3 co-immunostaining was performed in DLD-1 cells at 24 and 144 h post-treatment with DCB. Results showed a reduction in the frequency of cells displaying extra centrosomes over time (mean 65.96% at 24 h, mean 30.38% at 144 h after tetraploidization; *P* < 0.0001) (Supplementary Fig. 4a), thus revealing similar centrosome loss kinetics regardless of the mechanism used to generate tetraploidy.

To demonstrate that centriole reduction over time after tetraploidization is due to the loss of supernumerary centrioles in 4N cells rather than a decrease in the amount of the 4N subpopulation, we sought to determine the nuclear size in each time-point as a surrogate of nuclear DNA content. DAPI-stained nuclei were measured and revealed significant differences in the nuclear area between non-transfected and transfected DLD-1 2N cells (mean 82.29 μm^2^ *vs* 293.8 μm^2^; Mann-Whitney test, *P* < 0.0001) (Supplementary Fig. 4b); however, no significant differences in the nuclear area between different time-points after tetraploidization were found (mean 293.8 μm^2^ at 0 h, mean 291.7 μm^2^ at 24 h, mean 318.1 μm^2^ at 48 h, mean 282.4 μm^2^ at 96 h, mean 272.8 μm^2^ at 144 h after tetraploidization; ANOVA test, *P* = 0.19) (Fig. 6c), which suggested that cells spontaneously lost extra centrioles over time after becoming tetraploid.

Finally, we investigated the relationship between the frequency of newly-generated 4N cells harbouring extra centrioles and multipolar divisions. Our analysis indicated that the fraction of cells undergoing multipolar cell division was markedly lower than the fraction of cells exhibiting extra centrioles. Interestingly, the frequency of multipolar anaphases was significantly higher at 0 h after tetraploidization (35.25%, Tukey’s test, *P* < 0.0001), but no significantly differences were found across the other time-points (7.62% at 24 h, 5.64% at 48 h, 4.14% at 96 h, 2.70% at 144 h; ANOVA test, *P* = 0.52) (Fig. 6d). These results suggested that the clustering of supernumerary centrosomes in 4N cells occurred within the first 24 h after the generation of 4N cells to avoid multipolar mitosis, while the reduction of supernumerary centrioles took place progressively in the following cell divisions.

## Discussion

Tetraploidy, the state of having four sets of chromosomes, is a common transient intermediate state in cancer cells^28^. Tetraploid cells have shown greater tolerance to chromosome segregation errors, resulting in karyotypes with advantageous properties on cellular fitness. In addition, tetraploidy induces aberrant numbers of centrosomes, which promote merotelic kinetochore attachments and result in lagging chromosomes and aneuploidy. In this study, we analysed the centrosome dynamics in newly-generated tetraploid cancer cells as a consequence of silencing the protease separase or after DCB treatment, which hampers the separation of sister chromatids in anaphase or inhibits cytokinesis, respectively.

Tetraploid cells can originate by different mechanisms including cytokinesis failure, mitotic slippage, endoreduplication, or cell-to-cell fusion^29^. Regardless of these mechanisms, the resulting tetraploid cell will invariably contain extra centrosomes. Therefore, tetraploid cells must contain two centrosomes in G1 phase and centrosomes should be duplicated in the subsequent S phase^30^. Nevertheless, results obtained in the present study after surveying isogenic cancer 4N cells compared to their 2N counterparts, demonstrate that most of the cells maintained in culture contain only one centrosome in G1, thus reducing the chances of having multipolar cell divisions. Although the majority of cells show only one centrosome, a significant number of 4N cells still displays extra centrosomes, which could partially explain karyotype variability and elevated CIN^16^. The association of centrosome abnormalities with karyotype aberrations and disease progression strongly suggests centrosome amplification as a major underlaying cause of CIN in cancer^7,22,31^. Growing evidence indicates the importance of centrosome amplification in promoting tumorigenesis^10,12^, and its association with poor prognosis^32^. Moreover, a recent study has shown that supernumerary centrosomes induce the formation of invasive protrusions, which correlates with an invasive behavior^33^.

Besides alterations in the number of centrosomes, 4N cells maintained in culture did also show striking changes in the size of their centrosomes, resulting in enlarged pericentrin staining. Enlarged centrosomes can be originated by several mechanisms including (i) an increased amount of PCM^34^, (ii) having four or more centrioles as a consequence of PLK4 overexpression^35,36^, or (iii) as a result of centrosome clustering maintained in interphase^12,34,37^. In order to identify the actual origin of the enlarged centrosomes in 4N cells, centriole immunostaining was performed. Accordingly, although we did not immunoassayed with pericentrin and centrin3 simultaneously, the percentage of cells with four or more centrioles was higher in comparison to cells with two centrosomes, thus suggesting that cells with one enlarged pericentrin signal could indeed include extra centrioles. These results support the hypothesis that an enlargement in the size of the pericentrin labelling area is directly related to a higher number of centrioles, similarly to what other authors found in human mammary epithelial cells^38^, which leads to suggest that extra centrosomes clustered together and are maintained during G1-phase. A potential mechanism to explain the presence of supernumerary centrioles is due to an exceeding PLK4 activity^35^. However, overexpression of PLK4 in DLD-1 4N cells compared to their diploid counterparts was not detected by gene expression microarrays (data not shown). Taken together, our results indicate that oversized centrosomes in 4N cells could account for an excess number of centrioles per cell as a result of centrosome clustering.

Increased levels of CIN in tetraploid cells might be accompanied by changes in gene expression signatures. We therefore explored the intersection between deregulated genes in 4N cells and a CIN-associated gene expression signature^25^. Among those genes identified in common in both gene lists, we focused on *ESPL1*/Separase, which encodes the separase protein (also known as Separin). Separase overexpression has been shown in several tumour types compared to normal matched tissues^39–41^, and induces tumorigenesis in mice^42,43^. The protease separase has been implicated in several important biological processes. Separase has a central role in cell cycle progression by promoting the sister chromatid separation at anaphase onset. Thus, during normal mitotic progression, satisfaction of the spindle assembly checkpoint leads to securing ubiquitination and degradation by the anaphase-promoting complex/cyclosome (APC/C), separase activation, and the proteolytic cleavage of the SCC1/RAD21 subunit of the cohesion complex^27,44,45^. Together with PLK1, separase has also been implicated in promoting the loss of centriole tethering, a process known as disengagement, which license centrioles for their duplication^46^. Separase likely plays a supporting role in disengagement since loss of separase delayed assembly of new centrioles; however, most engagements were eventually dissolved, while PLK1 is essential to confer competence for re-duplication^4^. Previous reports have already shown that inhibition of the separase promotes tetraploidy, most likely due to cytokinesis failure^27,47,48^. Here, we find that the majority of newly-generated 4N cells obtained after silencing *ESPL1* contain two or more centrosomes, which contrasts with what is described in established 4N clones. Moreover, the time-course experiment to investigate the fate of extra centrosomes showed that centrosome loss occurs rapidly after the formation of tetraploidy. In fact, at 144 h after tetraploidization, the number of cells with four or more centrioles decreases to less than 25% of the cellular population, indicating that the amount of newly-generated 4N cells with supernumerary centrosomes after a prolonged culture is similar to that found in our established 4N cells *in vitro*. These results are in agreement with previous observations in non-transformed human cells suggesting that extra centrosomes are spontaneously lost while 4N cells divide in culture^22^. Here, we present robust data demonstrating that this loss also occurs in cancer cells independently of the *TP53* status (RKO is *TP53* wild-type, while DLD-1 is *TP53* mutated). Furthermore, DCB-treated cells display similar kinetics in supernumerary centrosomes reduction over time compared to *ESPL1*-depleted cells, revealing that the loss of extra centrosomes occurs regardless of the mechanism by which tetraploidy is induced.

The phenomenon of centrosome loss as an avenue to explain how polyploid tumour cells become stable and are able to expand has not been exploited in detail. The present study sheds light into how tetraploid cells are able to sustain cell division through the reduction of extra centrosomes. Different mechanisms to explain how tetraploid cells lose extra centrosomes over time might include centrosome elimination^49^, centrosome inactivation^50^, or selective clonal expansion of cells that have re-gained normal centrosome number^51^. Recent work by Baudoin and colleagues, considering tetraploid cells as those generated after a cytokinesis blockage in the binucleated stage, has proposed that asymmetric centrosome clustering in extra centrosome mitoses would result in the random generation of the fate of cell divisions with one centrosome, while extra centrosome cells undergo cell death or arrest^52^. However, our observations do not suggest high levels of cell death in newly-generated 4N cells. Therefore, further studies are needed to determine the most widespread mechanism to explain centrosome dynamics.

In summary, while most of the newly-formed 4N cancer cells rapidly lose extra centrosomes after passage in culture regardless of the mechanism by which tetraploidy is induced, a sub-fraction of cells is able to efficiently maintain the centrosome clustering during interphase, and therefore persist as a tetraploid population.

## Methods

### Cell culture and generation of clones

Colorectal cancer cell lines DLD-1, RKO and SW837 were obtained from the American Type Culture Collection (Manassas, VA, USA). DLD-1 and SW837 cells were cultured in RPMI1640 medium and RKO cells were cultured in DMEM/F-12 medium, both supplemented with antibiotics and 10% foetal bovine serum (Invitrogen, Carlsbad, CA, USA) at 37 °C in 5% CO_2_. Near-diploid (2N) and near-tetraploid (4N) clones of DLD-1 and RKO cell lines were previously generated in our laboratory^16^. Cytokinesis blockage with 1.5 μg/ml of dihydrocytochalasin B (Sigma-Aldrich, St. Louis, MO, USA) for 24 h was performed in double thymidine treated SW837 to establish 4N clones. Wild-type and post-tetraploid clones derived from the hTERT-immortalized retinal-pigmented epithelial cells (RPE1) (kindly provided by Z. Storchova, University of Kaiserslautern, Kaiserslautern, Germany) were cultured in DMEM/F-12 medium supplemented with antibiotics and 10% FBS at 37 °C in 5% CO_2_. For the experiments described in this study, we used one 2N clone and two 4N clones derived from the DLD-1, RKO and SW837 cell lines. As for the RPE1 cells, we used one 2N and one 4N clone.

### Transient reverse transfection

Two small interfering RNA (siRNA) molecules were used against *ESPL1* (Hs_ESPL1_5, 5′-CAGCAGCTGACTGCTAAGCTA-3′; Hs_ESPL1_6; 5′-TACCTCCAAGGTTAGATTTAA-3′) (Qiagen, Hilden, Germany) to generate transient silencing of this gene. Each siRNA at a final concentration of 5 nM was added to individual wells in a 6-well plate and complexed with lipofectamine RNAiMAX (ThermoFisher Scientific, Waltham, MA, USA) in serum-free medium for 30 min. Cells were then added in medium supplemented with 20% FBS to yield transfection mixtures in media containing 10% FBS. Final transfection mixtures were incubated at room temperature (RT) for 1 h before being placed at 37 °C in a humidified atmosphere containing 5% CO_2_. AllStars Hs Cell Death (Qiagen) was used as a positive cell death phenotype control, and AllStars Negative Control (Qiagen) was used as a negative control. Target-specific transfection efficiency was confirmed after 72 h at the mRNA level by real time RT-qPCR and after 96 h at the protein level by Western blot analysis. For both evaluations, mRNA and protein levels were compared with those found in cells transfected with negative control siRNA. Functional studies were conducted at 72 h post-transfection, except for cell viability analysis, which was conducted in 96-well plates at 96 h post-transfection and all reagent amounts were scaled down 30-fold.

### Metaphase spreads and fluorescence *in situ* hybridization (FISH)

Metaphase chromosomes were generated as previously described^53^. When required, cells were transfected for 72 h prior metaphase chromosomes were harvested. Metaphase spreads were captured using the Isis software v5.3 (Isis Fluorescence Imaging System, MetaSystems, Altlussheim, Germany; https://metasystems-international.com/en/products/isis/) on a Nikon Eclipse E50i microscope (Nikon Instruments, Tokyo, Japan). One hundred metaphase spreads were counted manually for each isogenic clone and each condition. Overlapping metaphase spreads were discarded.

The three-color centromeric FISH probes CEP4 Spectrum Green, CEP6 Spectrum Orange and CEP10 Spectrum Aqua (Abbott Molecular, Des Plaines, IL, USA) were used to quantify chromosomes 4, 6 and 10, respectively. For sample preparation and hybridization, a standard FISH protocol was used (https://ccr.cancer.gov/Genetics-Branch/thomas-ried). When required, FISH was performed after transfecting cells with siRNA for 72 h. Three hundred nuclei were analysed for each isogenic clone in each condition with a Nikon Eclipse E50i microscope. Interphase FISH images were acquired on the Isis software.

### Immunofluorescence

Cells seeded on coverslips were fixed for 15 min in 4% paraformaldehyde in PBS or PHEM buffer at RT followed by permeabilization with ice cold methanol at −20 °C or 0.5% of Triton X-100 in 1X PHEM at RT for 10 min. Fixed cells were then blocked with 5% NGS and 1% BSA in PBS or 10% BGS (20% BGS, 1X PHEM and DIH_2_O; 2:1:1) for 1 h, and incubated with primary antibodies diluted in blocking solution overnight at 4 °C. After primary antibody washing, secondary antibodies diluted in blocking solution were incubated for 45 min at RT. Cells were stained and mounted on microscope slides using ProLong Gold Antifade Mountant with DAPI (ThermoFisher Scientific). When required, cells were transfected with *ESPL1* siRNA (Qiagen) for 72 h followed by 0, 24, 48, 96 or 144 h of fresh media containing 10% FBS or treated with 1.5 µg/ml of DCB for 24 h followed by 24 or 144 h of fresh media containing 10% FBS, before performing the immunofluorescence. Immunostaining was performed using the following primary antibodies: rabbit anti-pericentrin (1:1000; Abcam, Cambridge, MA, USA), mouse anti-pericentrin (1:500; Abcam), mouse anti-centrin3 (1:200; Abnova, Taipei, Taiwan), mouse anti-cyclinD1 (1:100; Santa Cruz Biotechnology, Dallas, TX, USA), rabbit anti-cyclinD1 (1:100; Abcam) and mouse anti-ESPL1 (1:200; Abnova). Secondary antibodies were conjugated to Alexa Fluor 488 or Alexa Fluor 594 (both from ThermoFisher Scientific).

Immunofluorescence analysis was performed using a Nikon Eclipse E50i microscope. To determine the centrosome area, one hundred images of each isogenic clone were captured using a Nikon 100×1.30 NA oil objective and acquired by Isis software in areas of optimal cell density with minimal cellular clumps and overlapping cells. The centrosome area was quantified by ImageJ v1.50i (Image Processing and Analysis in Java, National Institutes of Health, Bethesda, USA; http://imagej.nih.gov/). To analyse separase fluorescence intensity, 40 images of each condition were captured with an exposure time of 1 ms using the same objective and software described above. Fluorescence intensities were measured using ImageJ.

### Microarray data

Gene expression microarray data were previously generated in our laboratory and normalized data can be extracted from the Gene Expression Omnibus database (National Centre for Biotechnology Information, Bethesda, MD, USA; https://www.ncbi.nlm.nih.gov/geo/) under accession number GSE81395. The Venn diagram was drawn through an online website http://bioinformatics.psb.ugent.be/webtools/Venn/.

### Quantitative real-time RT-PCR

One μg of total RNA isolated from cells with RNeasy Mini Kit (Qiagen) was used to generate cDNA using the High Capacity cDNA Reverse Transcription Kit (ThermoFisher Scientific) according to the manufacturer’s instructions. If needed, siRNA-mediated gene silencing was induced for 72 h before RNA extraction. Real-time PCR amplifications were performed in triplicate on 7300 Real-Time PCR System (ThermoFisher Scientific) using TaqMan Gene Expression Master Mix and TaqMan primers (both from ThermoFisher Scientific). Gene expression was normalized using *GAPDH*, and fold changes in mRNA expression were calculated using the 2^-ΔΔCt^ method.

### Lysate preparation and western blotting

Proteins were extracted from cultured cells with RIPA buffer (50 mM Tris-HCl, 1% NP-40, 0.5% Na-deoxycholate, 0.1% SDS, 150 mM NaCl, 2 mM EDTA, and 50 mM NaF, with protease inhibitors) to perform Western blot analysis. Protein samples were resolved by 6% acrylamide gels and electroblotted onto a PVDF membrane. The membrane was blocked in 3% BSA in tris-buffered saline, 0.1% Tween 20 (TBST) for 1 h, incubated with primary antibody diluted in blocking solution overnight at 4 °C, washed three times with TBST, incubated with secondary antibodies for 1 h at RT, and washed three times with TBST. For the detection of signals, SuperSignal West Femto (ThermoFisher Scientific) was used. Membranes were imaged on an ImageQuant LAS 4000 device (GE Healthcare Life Sciences, Chicago, IL, USA). The primary antibodies and dilutions used were: mouse anti–GAP120 (1:200; Santa Cruz Biotechnology), mouse anti-GAPDH (1:1000; Invitrogen) and rabbit anti-separase (1:200; Abcam). Blots were detected using goat anti-mouse and goat anti-rabbit (1:2500; ThermoFisher Scientific). ImageJ was used for quantification of the signal.

### Cell viability assay

CellTiter-Glo Luminescent Cell Viability Assay (Promega, Madison, WI, USA) was used according to the manufacturer’s instructions 96 h after siRNA transfection. Luminescence was measured after 10 min of incubation using a Synergy HT microplate reader (BioTek Instruments, Winooski, VT, USA). Viability of siRNA *ESPL1* transfected cells was compared to cells transfected with the negative control siRNA. Six replicates per each condition in two different plates were used to obtain measurements of cell viability.

### Nuclear area quantification

DAPI-based staining was used to measure the nuclear area in G1 phase cells. Cells were fixed in methanol-acid acetic (3:1), stained and mounted on microscope slides using Prolong Gold Antifade Mountant with DAPI, and imaged on an Olympus BX41 microscope (Olympus, Tokyo, Japan) equipped with a X100/1.25 NA oil objective. One hundred nuclei were analysed for each time-point. Studies were repeated in duplicate; overlapping cells were discarded. Nuclei images were captured with ZEN 2.6 software (Carl Zeiss, Jena, Germany; https://www.zeiss.com/microscopy/int/products/microscope-software/zen.html) and quantified by ImageJ software.

### Statistical analysis

Statistical analysis was performed using the software Prism v6 (GraphPad Software, San Diego, CA, USA; https://www.graphpad.com/scientific-software/prism/), and appropriate tests are indicated in the text and figure legends for each analysis when different from Student’s t-test.

## Supplementary information

Supplementary Figures

Supplementary Table 1.

## Data Availability

The data that support the finding of this study are available from the corresponding authors upon reasonable request.
