# Supplementary material for: Centrosome reduction in newly-generated tetraploid cancer cells obtained by separase depletion
Source: Sci Rep. 2020 Jun 4;10:9152. doi: 10.1038/s41598-020-65975-1 (PMC7272426; doi:10.1038/s41598-020-65975-1)

Centrosome reduction in newly-generated tetraploid cancer cells obtained by separase depletion

Claudia Galofré[1], Elena Asensio[1], Maria Ubach[2], Irianna M. Torres[1], Isabel Quintanilla[3], Antoni Castells[1], Jordi Camps[1,2,*]

1 Gastrointestinal and Pancreatic Oncology Team, Institut D'Investigacions Biomèdiques August Pi i Sunyer (IDIBAPS), Hospital Clínic de Barcelona, Centro de Investigación Biomédica en Red de Enfermedades Hepáticas y Digestivas (CIBEREHD), Barcelona (08036), Spain

2 Unitat de Biologia Cel·lular i Genètica Mèdica, Departament de Biologia Cel·lular, Fisiologia i Immunologia, Facultat de Medicina, Universitat Autònoma de Barcelona, Bellaterra (08193), Spain

3 Cell Biology of Genomes Group, National Cancer Institute, National Institutes of Health, Bethesda, MD (20817), USA

*Corresponding author:

Jordi Camps, Ph.D.

Gastrointestinal and Pancreatic Oncology Team

Institut D'Investigacions Biomèdiques August Pi i Sunyer (IDIBAPS)

Rosselló 149-153, 4th floor, 08036 Barcelona, Spain.

Phone: +34-93-2275400 ext.4560; Fax: +34-93-3129405

E-mail: jcamps@clinic.cat

SUPPLEMENTARY FIGURE LEGENDS

Figure S1. Assessment of karyotype heterogeneity. a,b) Dot plot depicting number of chromosomes in individual cells from one 2N and two 4N SW837 clones (a) and one 2N and one 4N RPE1 clones (b). Black lines denote median with interquartile range for each clone (n=100 metaphases/clone). **** represent $P<0.0001$.

Figure S2. Supernumerary centrosomes but no multipolar anaphases were found in 4N clones. a-d) Bar plots showing percentage of cells in G1 phase with their corresponding number of centrosomes for one 2N and two 4N clones of DLD-1 (a), RKO (b) and SW837 (c) cell lines, and for one 2N and one 4N RPE1 clones (d). A total of ~450 cells were analysed for each clone. Data are reported as mean ± SD. * represents $P<0.05$. e-h) Stacked bar graphs showing the frequency of bipolar and multipolar anaphases in 2N and 4N clones of DLD-1 (e), RKO (F), SW837 (g) and RPE1 (h) cell lines. No significant differences were found between 2N and 4N clones. More than 100 anaphases were analysed for each clone. Data are reported as mean.

Figure S3. Densitometry analysis and immunoblots of DLD-1 cells transfected with siRNA against *ESPL1*. a,b) Densitometry analysis (a) and the corresponding Western-Blot (b) of the full-length separase and the loading control GAP120 from the same gel after stripping the membrane (from Figure 4b). c,d) Densitometry analyses (c) of the full-length (top) and cleaved (bottom) separase and the corresponding Western-Blot (d). GAPDH was used as loading control after stripping the membrane.

Figure S4. Centrosome reduction in DCB-treated cells and nuclear area after siRNA transfection. Graph showing the number of centrioles at 24 and 144 h after

DCB treatment in DLD-1 cell line. A minimum of ~250 nuclei were analysed for each time-point. Data are reported as means ± SD. *** represent *P*<0.001, and **** *P*<0.0001 (n.s., not significant). b) Dot plot depicting nuclear area (μm^2) of DLD-1 2N cells untransfected and transfected with siRNA against *ESPL1* for 72 h. Data are indicated as mean ± SD (n=100 nuclei/ condition). **** represent *P*<0.0001.

Supplementary Fig. 1

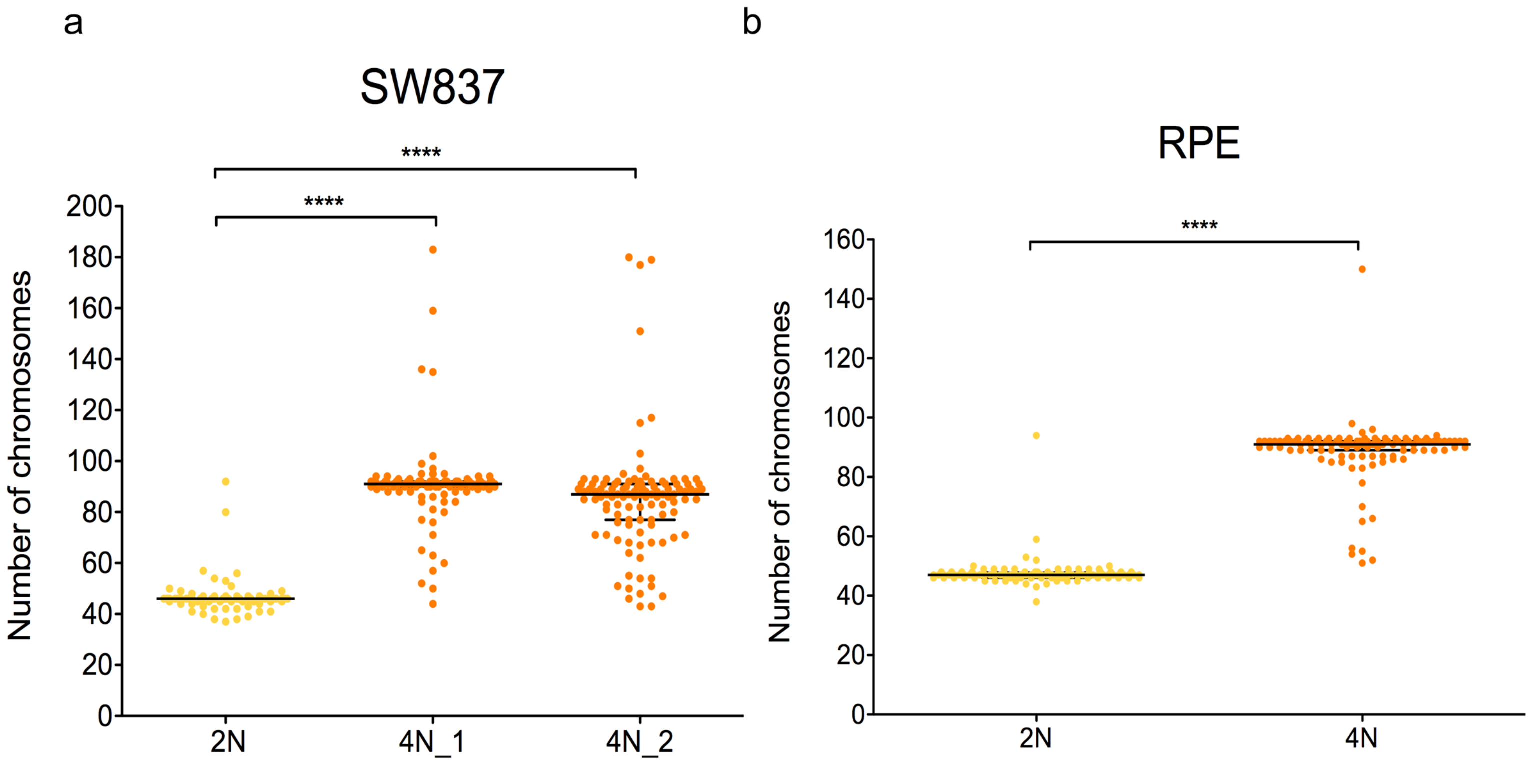

Supplementary Fig. 2

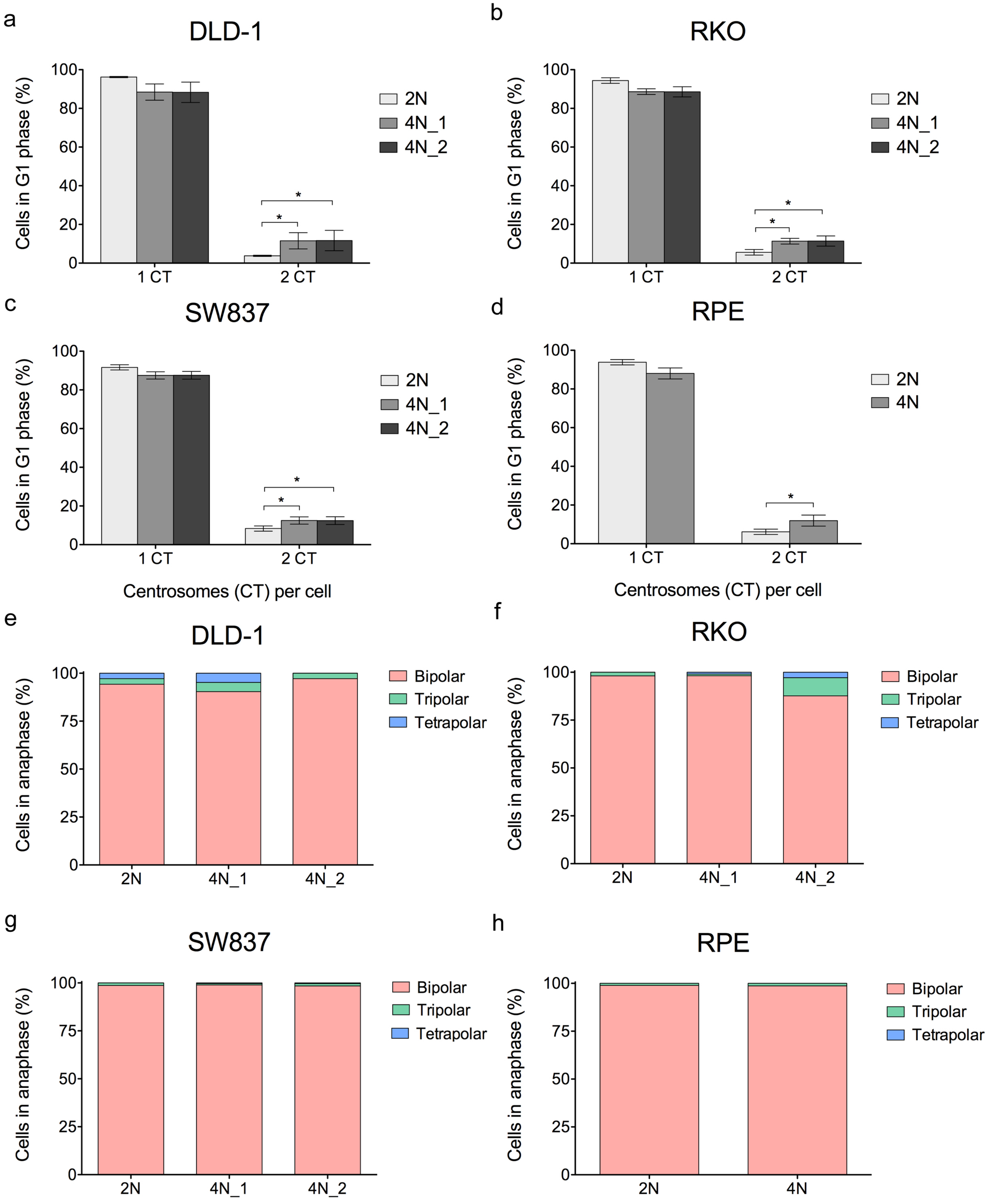

Supplementary Fig. 3

a

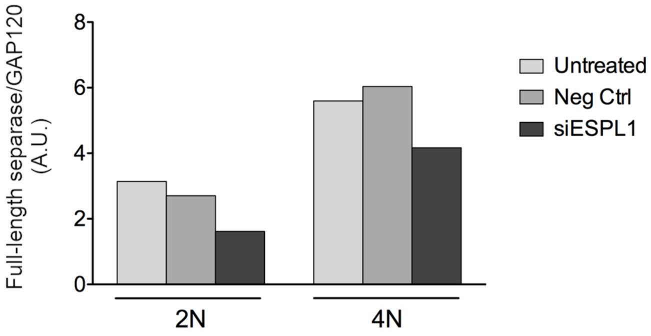

b

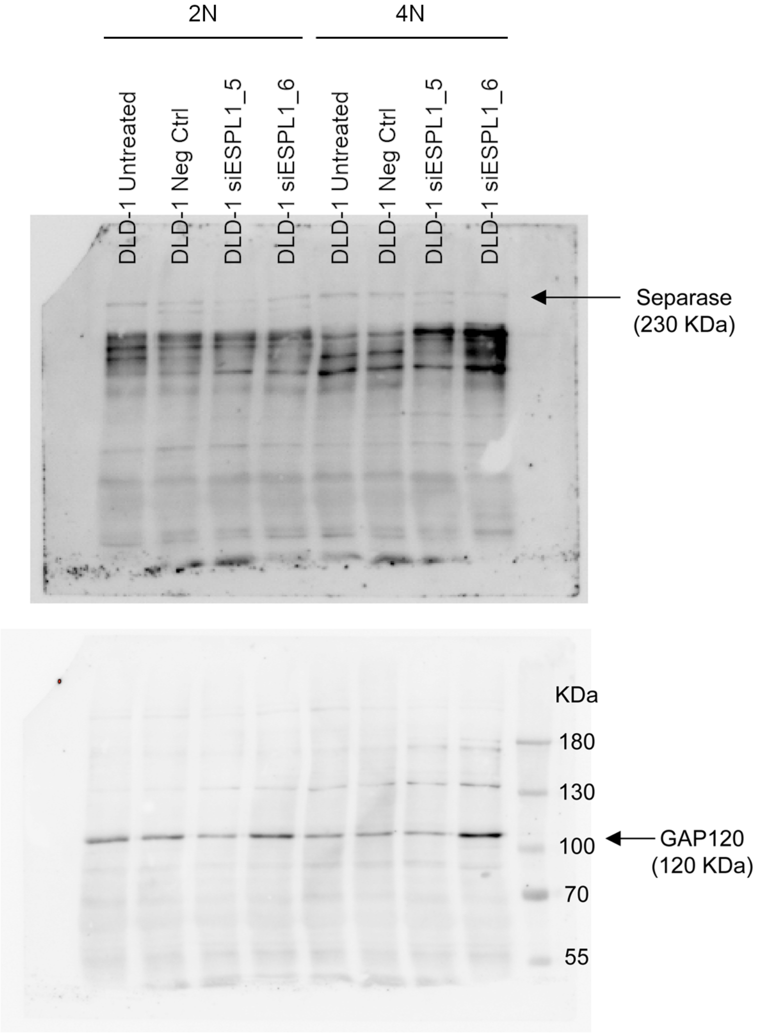

c

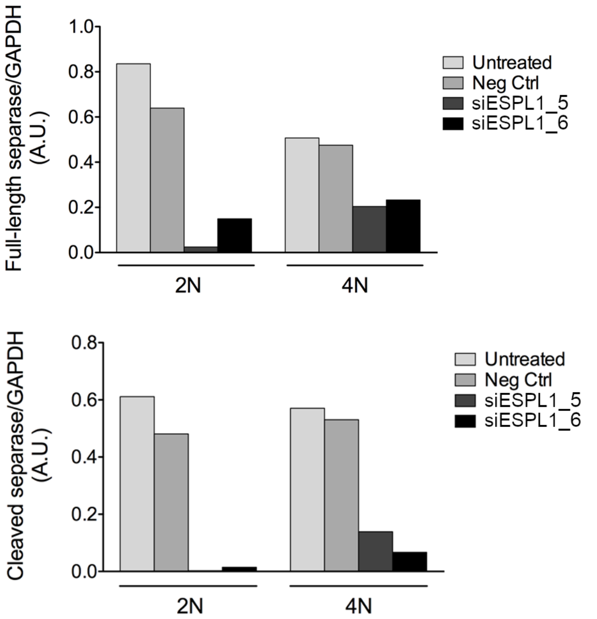

d

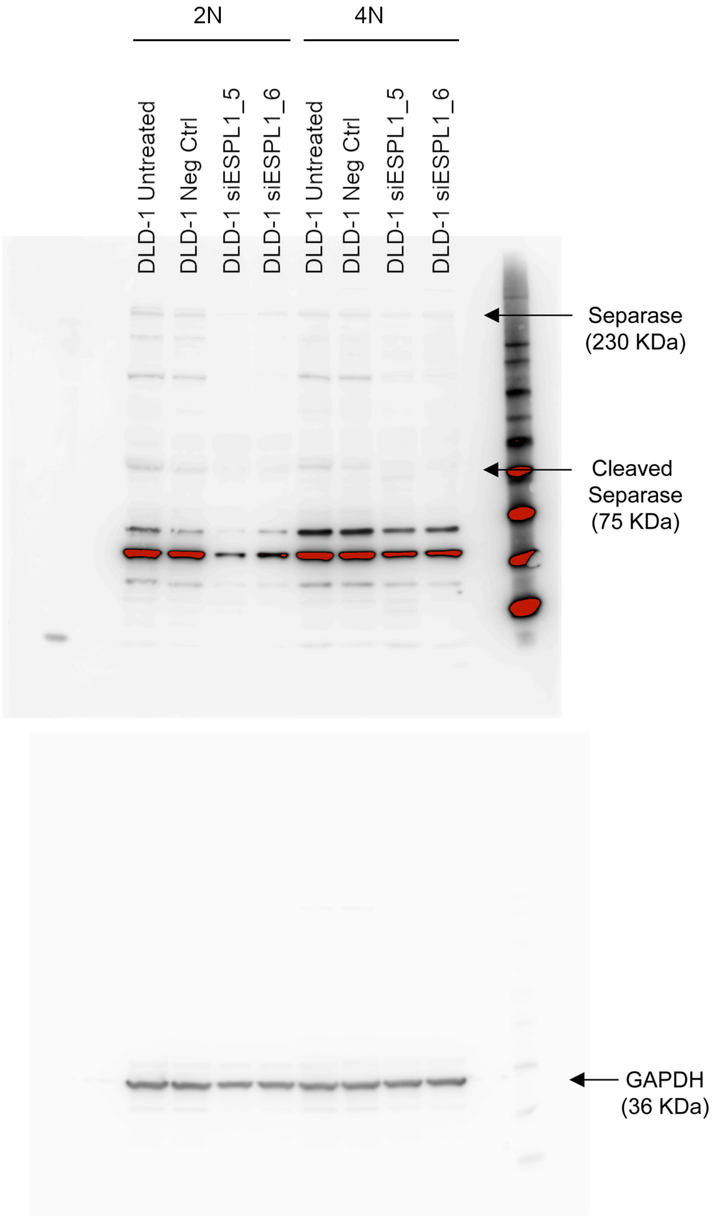

Supplementary Fig. 4

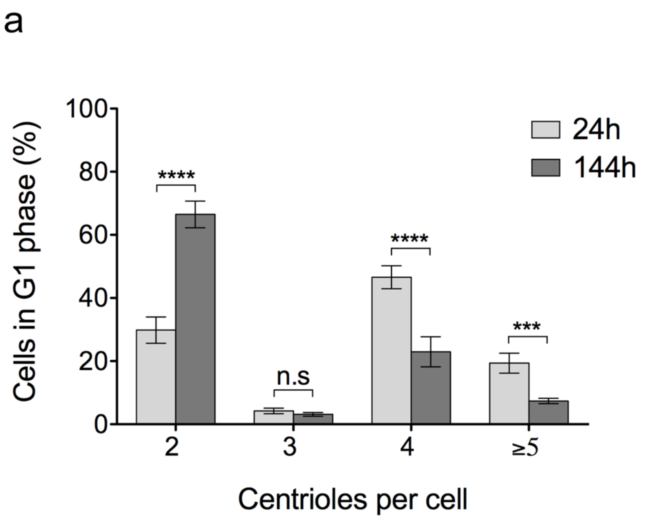

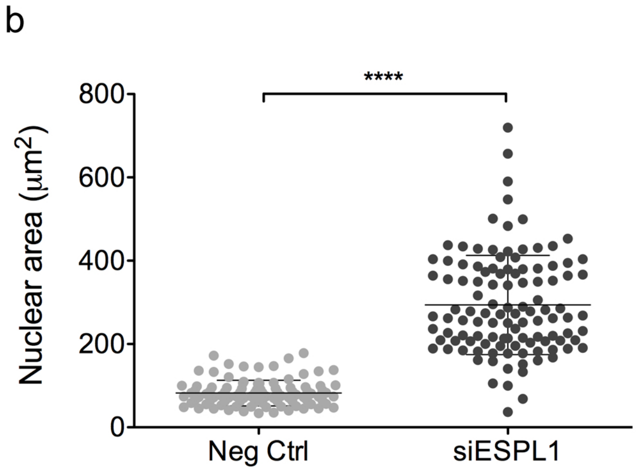

Supplement: Supplementary file 1 — Supplementary Figures [file 41598_2020_65975_MOESM1_ESM.pdf]
